# Supplementary figures and images for: Preliminary Identification of Candidate Genes Related to Survival of Gynogenetic Rainbow Trout (Oncorhynchus mykiss) Based on Comparative Transcriptome Analysis
Source: Animals (Basel). 2020 Jul 31;10(8):1326. doi: 10.3390/ani10081326 (PMC7459965; doi:10.3390/ani10081326)

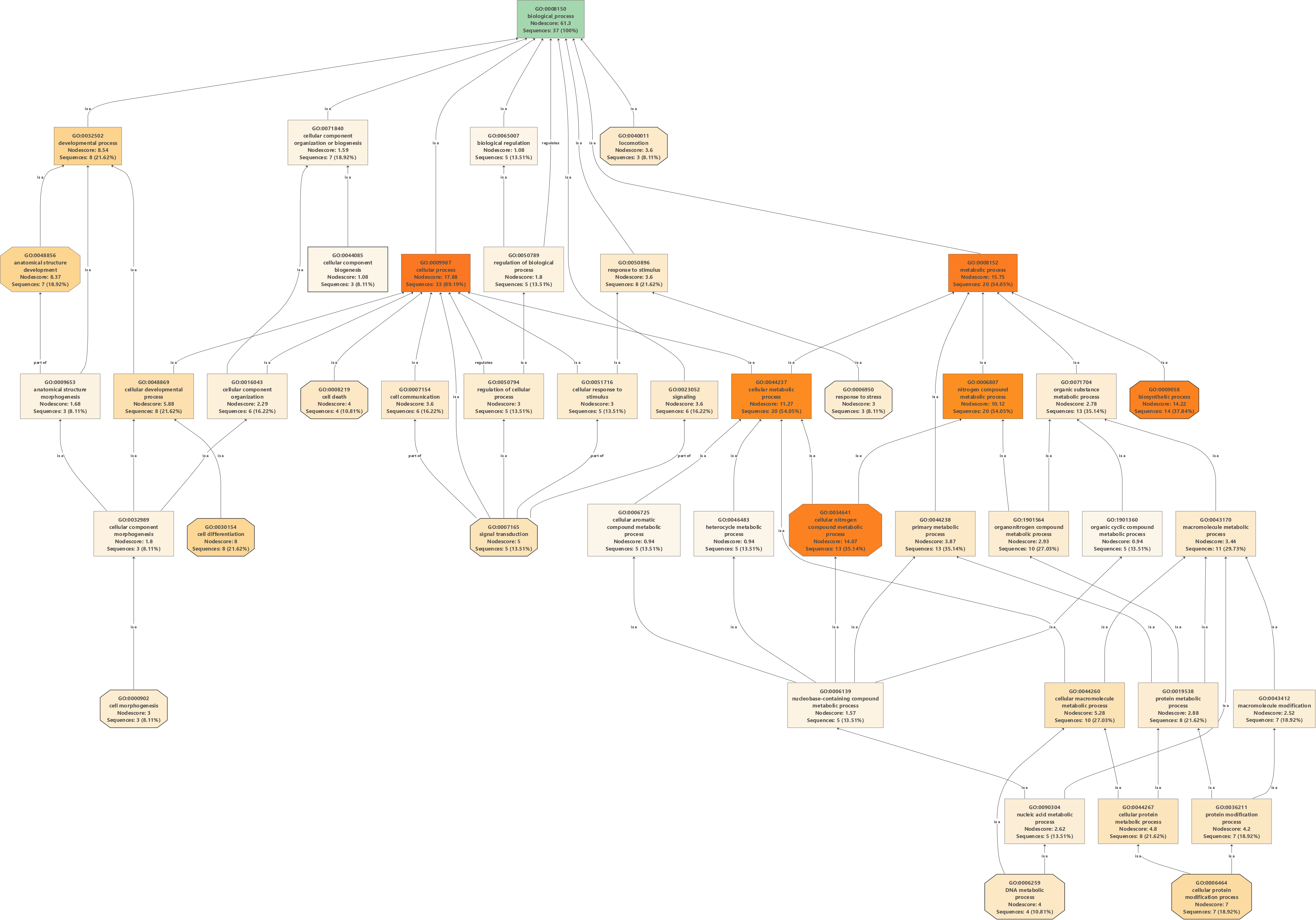

Supplement: Supplementary file 1 [file animals-10-01326-s001.zip › animals-844227-final-supplementary/Figure S1.png]

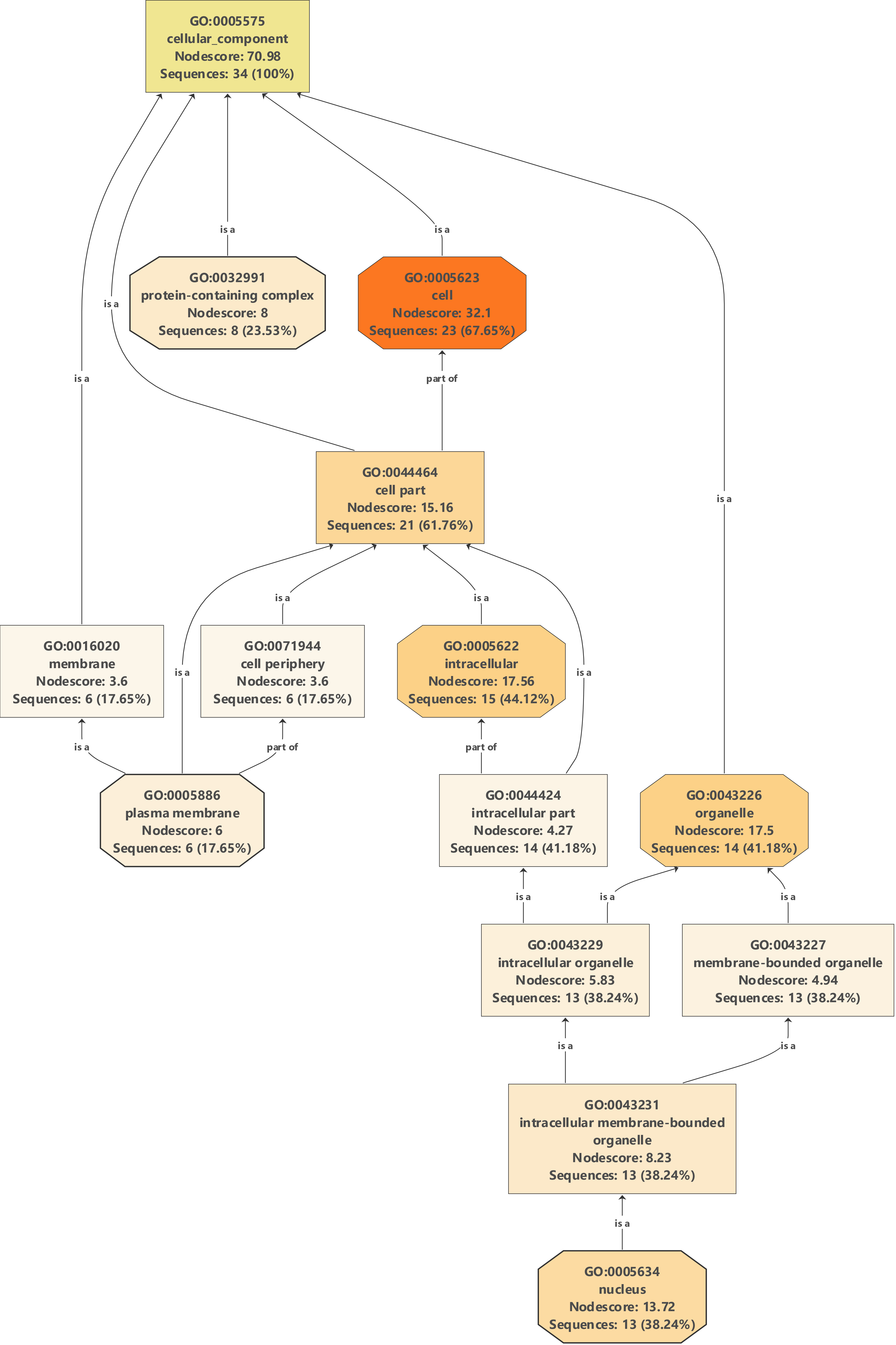

Supplement: Supplementary file 1 [file animals-10-01326-s001.zip › animals-844227-final-supplementary/Figure S2.png]

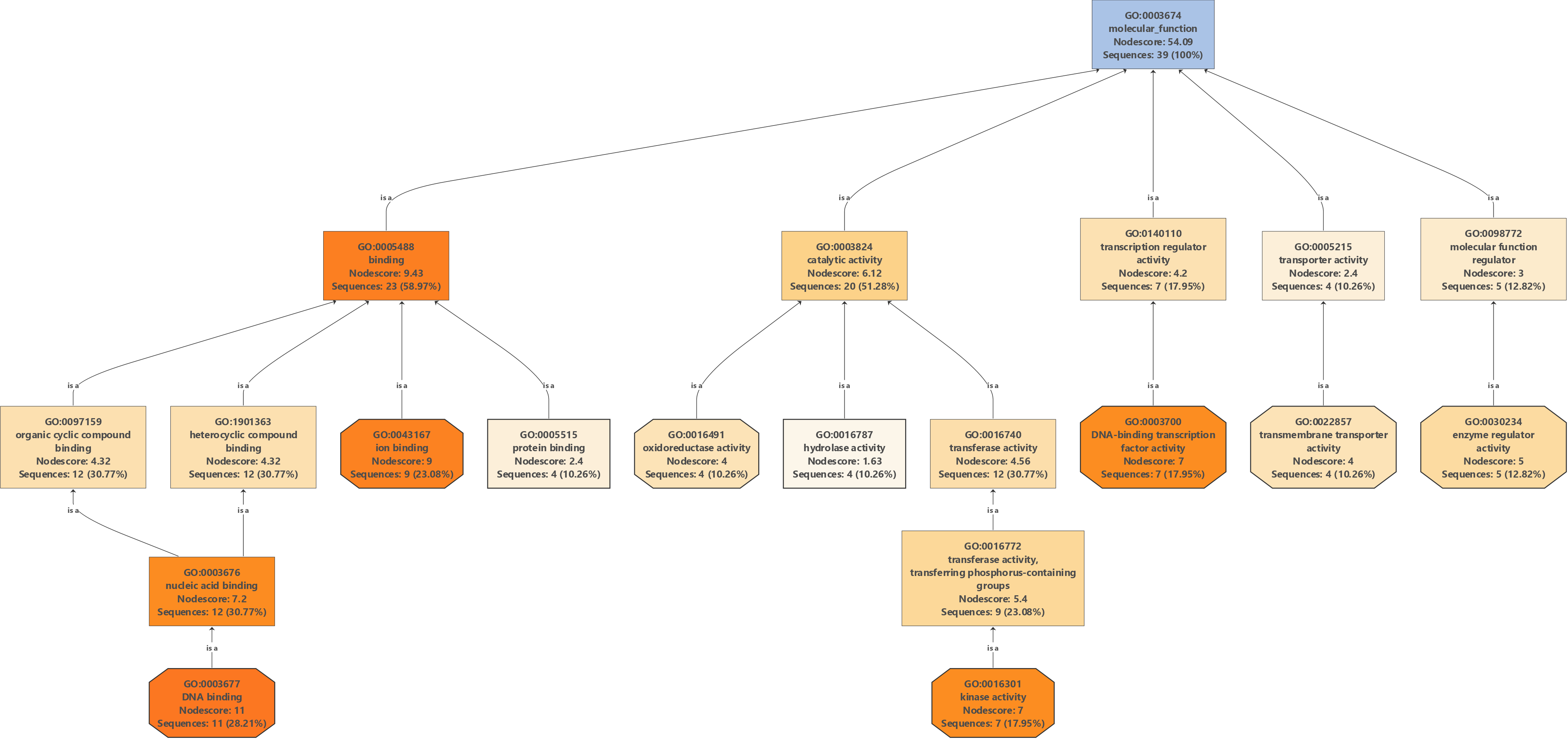

Supplement: Supplementary file 1 [file animals-10-01326-s001.zip › animals-844227-final-supplementary/Figure S3.png]
