# Supplementary material for: Preliminary Identification of Candidate Genes Related to Survival of Gynogenetic Rainbow Trout (Oncorhynchus mykiss) Based on Comparative Transcriptome Analysis
Source: Animals (Basel). 2020 Jul 31;10(8):1326. doi: 10.3390/ani10081326 (PMC7459965; doi:10.3390/ani10081326)
Supplement: Supplementary file 1 [file animals-10-01326-s001.zip › animals-844227-final-supplementary/Table S1.doc]

**Supplementary Table 1. Genotypes of the rainbow trout egg donors and gynogenetic larvae.**

| **Eggs donors** | **Locus** | **Maternal Genotype** | **Genotype of gynogenetic progenies** | **Number of inviduals** |
| --- | --- | --- | --- | --- |
| 1 | OMM 1101 | 190/200 | 190/190 | 5 |
|  |  |  | 190/200 | 0 |
|  |  |  | 200/200 | 2 |
|  | OMM 1127 | 180/220 | 180/180 | 5 |
|  |  |  | 180/220 | 0 |
|  |  |  | 220/220 | 2 |
|  | OMM 1131 | 290/310 | 290/290 | 5 |
|  |  |  | 290/310 | 0 |
|  |  |  | 310/310 | 2 |
| 2 | OMM 1025 | 320/380 | 320/320 | 8 |
|  |  |  | 320/380 | 0 |
|  |  |  | 380/380 | 7 |
|  | OMM 1053 | 240/260 | 240/240 | 9 |
|  |  |  | 240/260 | 0 |
|  |  |  | 260/260 | 6 |
|  | OMM 1075 | 250/270 | 250/250 | 6 |
|  |  |  | 250/270 | 0 |
|  |  |  | 270/270 | 9 |
|  | OMM 1101 | 190/200 | 190/190 | 9 |
|  |  |  | 190/200 | 0 |
|  |  |  | 200/200 | 6 |
|  | OMM 1107 | 200/230 | 200/200 | 10 |
|  |  |  | 200/230 | 0 |
|  |  |  | 230/230 | 5 |
|  | OMM 1238 | 280/400 | 280/280 | 5 |
|  |  |  | 280/400 | 0 |
|  |  |  | 400/400 | 10 |
| 3 | OMM 1053 | 220/290 | 220/220 | 6 |
|  |  |  | 220/290 | 0 |
|  |  |  | 290/290 | 5 |
|  | OMM 1084 | 210/290 | 210/210 | 6 |
|  |  |  | 210/290 | 0 |
|  |  |  | 290/290 | 5 |
|  | OMM 1238 | 280/310 | 280/280 | 4 |
|  |  |  | 280/310 | 0 |
|  |  |  | 310/310 | 7 |
| 4 | OMM 1053 | 210/290 | 210/210 | 11 |
|  |  |  | 210/290 | 0 |
|  |  |  | 290/290 | 4 |
|  | OMM 1084 | 220/280 | 220/220 | 5 |
|  |  |  | 220/280 | 0 |
|  |  |  | 280/280 | 10 |
|  | OMM 1238 | 320/450 | 320/320 | 7 |
|  |  |  | 320/450 | 0 |
|  |  |  | 450/450 | 8 |
